# Procrastination among students of Polish universities. Protective and predisposing factors

**DOI:** 10.1192/j.eurpsy.2023.774

**Published:** 2023-07-19

**Authors:** J. A. Smolarczyk-Kosowska, P. Dębski, A. Ngo, M. Piegza

**Affiliations:** 1 Department of Psychoprophylaxis Medical University of Silesia; 2 Department of Psychiatry Medical University of Silesia, Medical University of Silesia, Tarnowskie Góry, Poland

## Abstract

**Introduction:**

Procrastination is a widely recognized phenomenon that can be defined as a tendency to delay important life activities and decisions. This seems to be a common issue among schoolchildren and students, however is not limited to those populations and may also be observed among other social groups. Research shows that the indices of the occurence of procrastination may reach even as much as 20-25% of the total population. However, the frequency of the phenomenon seems to be considerably higher among academic environment.

**Objectives:**

We sought to investigate the association between the phenomenon of procrastination and the individual’s resiliency, sense of coherence and the development of the identity.

**Methods:**

A web-based questionnaire study was performed on a random sample of Polish students of various fields of study, aged 18-30 years. A 131-item questionnaire was built based on an original questionnaire and four standardized tools for the assessment of procrastination tendencies, resiliency, sense of coherence and the development of the identity (polish-Kwestionariusz Zwlekania-KZ), the Sense of Coherence Questionnaire-SOC-29, the Ego Resiliency Scale, The Dimensions of Identity Development Scale- DIDS).

**Results:**

This questionnaire study included 294 participants, median age 22 (IQR=21-24). Mature forms of identity development (meaning the commitment making (CM) and identification with commitment (IC)) correlated negatively with the occurence of procrastination (R=-0,186, p<0.005 for CM; R=-0.288, p<0.05 for IC). Ruminative exploration (RE) correlated positively with tendency to procrastinate (R=0.218, p<0.05). Procrastination correlated negatively with the resiliency evaluation (R=-0.229, p<0.05) and the optimal regulation(OR) assessment (R=-0.255, p<0.05). All the SOC-29 domains along with its total score correlated negatively with the tendency to procrastinate (p<0.05 for all).

**Conclusions:**

Based on the conducted study, there seem to be measurable psychological benefits regarding the individual’s personal performance resulting from a proper psychoeducation in the field of procrastination.

**Disclosure of Interest:**

None Declared

